# The Essentials of Pathology

**Published:** 1883-10

**Authors:** 


**The Essentials of Pathology.** By D. TOD GILLIAM, M. D., Professor of Physiology, Starling Medical College, formerly Professor of General Pathology, Columbus Medical College, Columbus, Ohio. Philadelphia: P. Blakiston, Son & Co. 1883.

The author of this compilation states that the object of the work is "to unfold to the beginner the fundamentals of pathology in a plain, practical way," and we must admit that he has fairly accomplished his purpose. The value of the book is increased by numerous admirable illustrations, which look very familiar, and a comparison shows that they are largely taken from the classical work of Rindfleisch. It would have been only fair to have acknowledged this in the text, but they not the less greatly add to the value of the work. We think such a work as this was needed, and we predict for it a large sale.

